# Fast Star Matching Method Based on Double *K*-Vector Lookup Tables for Multi-Exposure Star Trackers

**DOI:** 10.3390/s21093176

**Published:** 2021-05-03

**Authors:** Wenbo Yu, Jie Jiang, Pei Wu, Chuanzhong Xuan, Chunhui Zhang

**Affiliations:** 1 College of Mechanical and Electrical Engineering, Inner Mongolia Agricultural University, No. 306 Zhaowuda Road, Saihan District, Hohhot 010018, China; hityuwenbo@imau.edu.cn (W.Y.); jdwp@imau.edu.cn (P.W.); xcz@imau.edu.cn (C.X.); Zhch1979@126.com (C.Z.); 2 Key Laboratory of Precision Opto-Mechatronics Technology, Ministry of Education, School of Instrumentation Science and Opto-Electronics Engineering, Beihang University, No. 37 Xueyuan Road, Haidian District, Beijing 100191, China

**Keywords:** high update rate, multi-exposure imaging, star tracker, double *K*-vector lookup tables, fast star matching

## Abstract

A high update rate is always one of the vital indices of star trackers. By recording star positions at *N* moments within a single star image, the multi-exposure imaging approach (MEIA) proposed in an earlier study can improve the attitude update rate of star trackers by *N* times. Unfortunately, when the existing star matching method is adopted to match the observed and predicted stars in MEIA, the matching time is significantly increased with the increase in multi-exposure times, *N*, or the number of navigation stars, *M*, which sharply affects the MEIA’s performance. Therefore, a fast star matching method based on double *K*-vector lookup tables (DKVLUTs) is proposed to address the above issue. In this method, the information of all predicted stars is used to establish the DKVLUT, and thus, the speed of the whole matching process between observed and predicted stars would be increased effectively by means of the DKVLUT. Both simulations and experiments are conducted to verify the performance of the proposed method. The results both show that the matching time of the proposed method is reduced by nearly one order of magnitude compared with that of the existing method, which demonstrates the feasibility and effectiveness of the proposed method.

## 1. Introduction

A star tracker is a kind of celestial navigation equipment, which has the advantages of low power consumption, high precision, no drift and autonomy; as such, it has been widely used in the field of aeronautics and astronautics [1,2,3,4,5,6]. The attitude update rate of traditional star trackers is relatively low, and it is generally around 4–10 Hz. With the continuous advancement of aeronautics and astronautics, some navigation tasks, such as those for agile satellites, space weapons or ballistic missiles, etc., have high requirements for rapid maneuverability of the carrier [7,8,9]. As the maneuverability of the carrier increases, the change rate of the attitude increases accordingly. According to the sampling theorem, the attitude update rate of a star tracker should be increased synchronously so as to achieve an accurate description and effective recovery from discrete sampling data to continuously changing attitude data. Therefore, having a high update rate has become quite an important development direction for star trackers [10].

Many researchers have carried out several approaches to effectively improve the attitude update rate of star trackers. Firstly, the integrated navigation system of the star tracker and an inertial measurement unit is a classic scheme to improve the attitude update rate of the whole system [11,12,13,14]. Moreover, when the three-field-of-view scheme is adopted to the star tracker, the overall attitude update rate can reach up to three times that of a single-field-of-view one [15,16]. However, the above two approaches both have the problems of large volume, great weight and high power consumption, which are not conducive to the light weight and miniaturization of products [17,18,19]. By contrast, the approach to improving the attitude update rate of the single-field-of-view star tracker has more extensive application value. Zhong et al. [20] and Mao et al. [21] adopted a parallel and pipeline processing method to improve the attitude update rate. They generally divided the whole working process of star trackers into four stages: exposure, transmission and processing of pixels, star matching and attitude calculation. Among them, the steps of star matching and attitude calculation usually take relatively less time, while high-sensitivity image detectors can also reduce the exposure time to some extent. Therefore, the time of transmission and processing is the major bottleneck of the attitude update rate. 

In order to break through the above bottleneck and further improve the attitude update rate, a multi-exposure imaging approach (MEIA) for star trackers was proposed by the authors in an earlier study [22]. In this approach, *N* short-time exposures can be adaptively inserted into a single star image according to the angular velocity of the star tracker, and thus, *N* different groups of star positions would be recorded in the above multi-exposure star image. When the attitude calculation is carried out at the *N* imaging moments, *N* corresponding groups of attitude information can be obtained, which is equivalent to increasing the attitude update rate by *N* times in theory. It should be noticed that the number *N* cannot be set arbitrarily, but should increase with the increase in the angular velocity of the star tracker to avoid overlapping of the adjacent star points. Simulation results [22] show that the MEIA’s attitude update rate can reach up to 210 Hz with the angular velocity increasing to 20 °/s, which is much higher than the 10-hertz update rate of the traditional single-exposure imaging method. 

Nevertheless, with the increase in *N*, the total number of star points within a multi-exposure star image increases sharply. On the one hand, if all observed star points are directly matched with all predicted star points one by one (the existing matching method that is widely used in traditional area of star trackers), the complexity and the time of the star matching step will be significantly increased, thus resulting in the star matching step becoming the new bottleneck of the MEIA’s attitude update rate. On the other hand, although the mainstream feature points-based image registration methods are quite popular and successful in many applications [23,24,25], they are not applicable for the above star matching situation. This is because the above matching methods are mainly used to solve the point matching problem between two real images (i.e., the matching image and the matched image), while the star matching step only contains one real image (i.e., the observed star image) and one dataset of the centroid information of the predicted star points. 

Considering this, a fast star matching method based on a double *K*-vector lookup table (DKVLUT) is proposed in this paper. Firstly, according to the *x*-coordinates and *y*-coordinates of all the predicted stars, the DKVLUT for both ***K****_x_* and ***K****_y_* can be established. Secondly, when the actual observed star point is obtained by scanning the image, an appropriate searching range, which is preset according to the precision of the star prediction, is utilized to search the ***K****_x_* and ***K****_y_*, and the intersection of the above two searching results is the final matching result. In essence, the proposed method is a kind of matching method between two association datasets rather than an image pixel feature-based matching method.

The remainder of this paper is organized as follows. The problems of the existing star matching method are analyzed in detail in Section 2. Section 3 details the principle of the proposed fast star matching method based on DKVLUTs. Simulation experiments and real night sky experiments are conducted and detailed in Section 4 and Section 5, respectively, to demonstrate the feasibility and effectiveness of the proposed method. Section 6 presents a discussion on the proposed method. Finally, conclusions are drawn in Section 7. 

## 2. Problems of the Existing Star Matching Method

**Figure 1:**
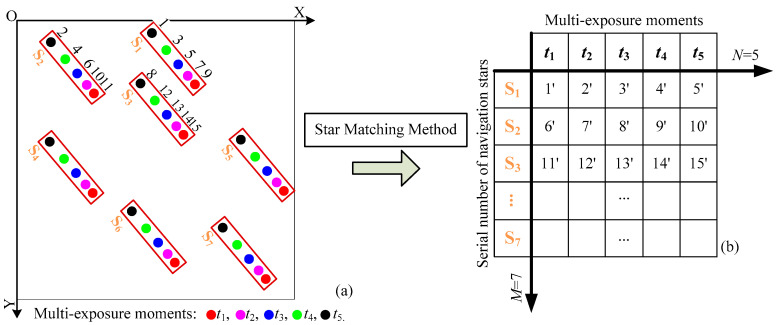
Scanning sequence and grouping sequence of the multi-exposure star image. (**a**) Scanning sequence of observed star points; (**b**) grouping sequence of predicted star points.

The MEIA’s partial schematic star image is shown in Figure 1a. The multi-exposure times and the number of navigation stars in the schematic star image are set as *N* = 5 and *M* = 7, respectively, and then, the star image contains the centroid information of *W* = *NM* = 35 star points altogether. When the star tracking and attitude estimation are carried out at the *N* imaging moments, *N* corresponding groups of attitude information can be obtained, which is equivalent to increasing the attitude update rate by *N* times. 

However, as is shown in Figure 1a, when scanning the multi-exposure star image, the actual centroids of the observed star points at different positions are obtained in sequence from left to right and from top to bottom in the star image. Therefore, all the actual centroid information belonging to *M* different navigation stars at *N* different sampling moments presents a kind of disordered distribution. By contrast, when the theoretical centroids of the predicted star points are accurately predicted according to the attitude, angular velocity and angular acceleration [26], all the theoretical centroid information belonging to *M* different navigation stars at *N* different sampling moments presents a kind of sequential distribution, which is shown in Figure 1b. In Figure 1, the numbers 1, 2, 3, etc., indicate the serial numbers of observed star points, while the numbers 1′, 2′, 3′, etc., indicate the serial numbers of predicted star points. When taking star S_1_ as an example, it is necessary to match the observed star points (1, 3, 5, 7, 10) in Figure 1a with the predicted star points (1′, 2′, 3′, 4′, 5′) in Figure 1b one by one. When all observed star points are correctly matched, the centroid information of observed star points at *N* different sampling moments can be used for star tracking and attitude calculation, thus improving the update rate of MEIA. 

The existing star matching method [27] usually directly matches all observed star points in Figure 1a with all predicted star points in Figure 1b one by one. When the observed star point to be matched and a certain predicted star point meet the requirement of the star neighborhood approach (SNA), it indicates that the matching is successful. The same matching process is repeated for all observed star points until all of them are matched successfully. Nevertheless, since the multi-exposure star image contains the star points belonging to *M* different navigation stars at *N* different sampling moments, the total number of star points increases significantly compared to the traditional single-exposure star image. In the worst case scenario, the existing star matching method may need to repeat the matching process *W*! = (*MN*)! times at most. With the increase in *M* or *N*, the total matching times, W!, increase sharply, and thus, the matching time increases significantly, which is not beneficial to MEIA’s application. 

## 3. Principle of the Fast Star Matching Method 

**Figure 2:**
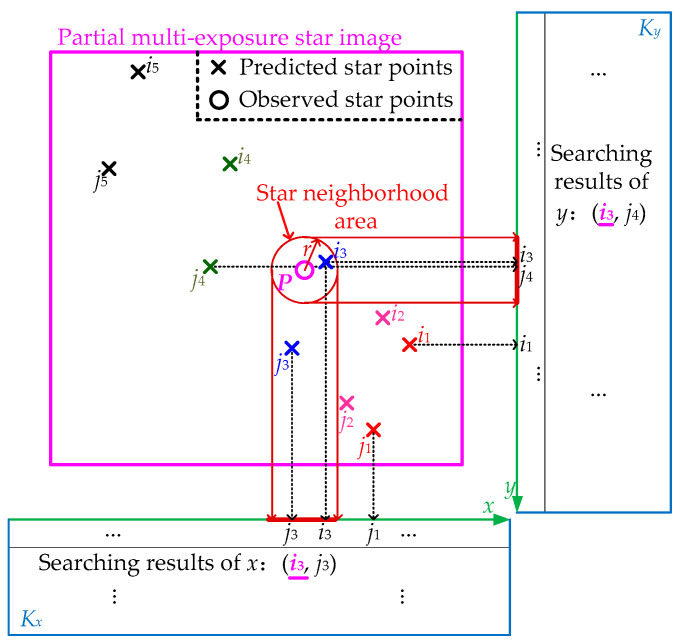
Basic principle of the fast star matching method based on a DKVLUT.

In order to speed up the star matching process and reduce the matching time for the multi-exposure star image, a fast star matching method based on a double *K*-vector lookup table (DKVLUT) is proposed in this paper. The method based on *K*-vector lookup tables is actually a kind of curve fitting technology, which can realize quick searching among lots of elements by utilizing the monotone increasing function. Figure 2 shows the basic principle of the proposed star matching method. For the convenience of expression, only a partial multi-exposure star image, which contains the star points of *M* = 2 navigation stars at *N* = 5 sampling moments, is illustrated in Figure 2. The predicted star points, which are numbered *i*_1_~*i*_5_ and *j*_1_~*j*_5_, are accurately obtained by using the attitude, angular velocity and angular acceleration of the star tracker [26].

In Figure 2, star point *P* is one of the actual observed star points in the current multi-exposure star image, and *r* represents the radius of the star neighborhood area, which is determined by the accuracy of star prediction. The whole matching process of star point *P* is described as follows: Firstly, the centroid information of all predicted star points is divided into two parts, *x*-coordinates and *y*-coordinates, which need to be arranged from small to large, and then, the DKVLUT for both ***K****_x_* and ***K****_y_* can be established according to the above sequential *x*-coordinates and *y*-coordinates, respectively. Secondly, when star point *P* is obtained by scanning, its star neighborhood area, whose size is determined by *r*, will be utilized to search in ***K****_x_* and ***K****_y_*. The search results of ***K****_x_* and ***K****_y_* are (*i*_3,_ *j*_2_) and (*i*_3_, *j*_5_), respectively, and thus, the intersection of the above two search results, *i*_3_, is the final matching result of star point *P*, which is consistent with the correct match result shown in Figure 2. In summary, the whole matching process can be mainly divided into two steps, i.e., the establishment of the DKVLUT and the matching of the observed star points, which will be detailed below. 

### 3.1. Establishment of the DKVLUT

**Figure 3:**
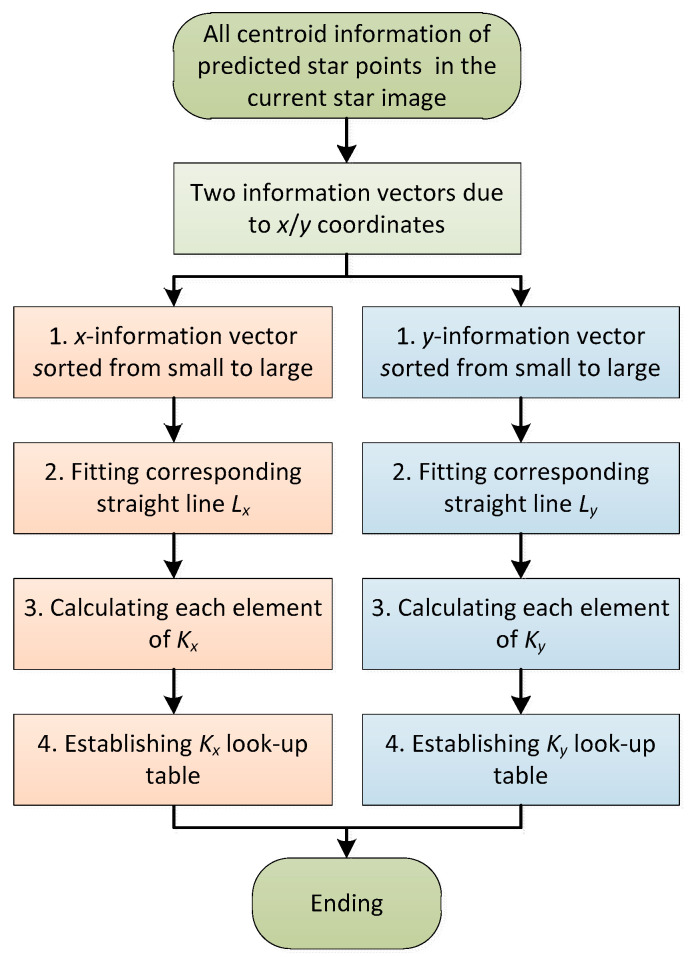
Flow chart of the establishment of the DKVLUT.

Figure 3 is the flow chart of the establishment of the DKVLUT. According to the attitude and the complete motion parameters (i.e., the angular velocity and the angular acceleration) obtained from the previous multi-exposure star image [26], all the centroid information of the predicted star points corresponding to each sampling moment in the current multi-exposure star image can be accurately estimated. The centroid information of each predicted star point is composed of an *x*-coordinate and *y*-coordinate, and thus, all the centroid information of the predicted star points can be divided into two information vectors, ***S****_x_* and ***S****_y_*, whose lengths are both *l_s_* = *W.* From a mathematical point of view, the establishment processes of the DKVLUT for both ***K****_x_* and ***K****_y_* are exactly the same, so the following deduction will only take the establishment process of ***K****_x_* as an example. 

Firstly, since the monotone increasing function is utilized to fulfill the fast search in the DKVLUT, the information vector, ***S****_x_*, has to be rearranged from small to large according to the *x*-coordinate. Meanwhile, the rearrangement of ***S****_x_* also results in the rearrangement of the serial numbers of all predicted star points, and the serial number vector after rearrangement can be defined as ***R****_x_*. After the rearrangement, the information vector, ***S****_x_*, has the monotone increasing characteristic, which can be described as ***S****_x_*(*j*) ≤ ***S****_x_*(*j* + 1) where *j* is the serial number of the information vector element and satisfies 1 ≤ *j* ≤ *l_s_* −1. Obviously, the two endpoints of ***S****_x_* are (1,***S****_x_*(1)) and (*l_s_*,***S****_x_*(*l_s_*)), and (*l_s_* − 1) intervals are totally included between the above two endpoints. The average distance of each interval, *D_x_*, can be expressed as follows:

(1) $$D_{x}=\frac{S_{x}(l_{s})-S_{x}(1)}{l_{s}-1}.$$

Then, in order to fully contain the two endpoints of ***S****_x_*, two actual points, A (1, ***S****_x_*(1) − *D_x_*/2) and B (*l_s_*, ***S****_x_*(*l_s_*) + *D_x_*/2), are both used to fit a line, *L_x_*, whose expression is as follows:

(2) $$h_{x}=a_{1}\cdot m_{x}+b_{1},$$

where *m_x_* and *h_x_* are the independent and dependent variables of the fitting line, *L_x_*, respectively; and *a*_1_ and *b*_1_ are the parameters of the fitting line, *L_x_*, respectively, whose expressions can be described as follows:

(3) $$a_{1}=\frac{l_{s}\cdot D_{x}}{l_{s}-1},b_{1}=S_{x}(1)-\frac{D_{x}}{2}-a_{1}.$$

Finally, according to the fitting line, *L_x_*, as well as the information vector, ***S****_x_*, all elements of ***K****_x_* can be calculated one by one, and the corresponding calculation rules are described as follows:

(1) ***K****_x_* is a one-dimensional integer vector, and its length is the same as that of ***S****_x_*, which are both *l_s_*.

(2) The starting and ending elements of ***K****_x_* are ***K****_x_*(1) = 0 and ***K****_x_*(*l_s_*) = *l_s_*, respectively. 

(3) The other elements of ***K****_x_* should satisfy ***K****_x_*(*i*) = *j* when ***S****_x_*(*j*) ≤ *h_x_*(*i*) < ***S****_x_*(*j* + 1), in which the serial numbers of ***K****_x_* and ***S****_x_*, i.e., *i* and *j*, are from 2 to (*l_s_* − 1) and from 1 to (*l_s_* − 1), respectively. 

In essence, the physical meaning of ***K****_x_* lies in that the *i*th (1 ≤ *i* ≤ *l_s_*) element of ***K****_x_* represents the number of elements of ***S****_x_* that are less than or equal to the function value *h_x_*(*i*) of the fitting line *L_x_*. 

Based on the above rules, all elements of ***K****_x_* can be calculated one by one, and the corresponding elements of ***S****_x_* and ***R****_x_* can also be listed. Furthermore, ***U****_x_* represents the line number vector of ***K****_x_*, and all information vectors including ***K****_x_*, ***S****_x_*, ***R****_x_* and ***U****_x_* are shown in Table 1. Similarly, for the *y*-coordinate, the same process as the *x*-coordinate mentioned above can be adopted, and the four corresponding information vectors are ***K****_y_*, ***S****_y_*, ***R****_y_* and ***U****_y_*. In summary, the DKVLUT of all predicted star points in the current frame, both ***K****_x_* and ***K****_y_*, can be established according to the above steps. 

### 3.2. Matching of the Observed Star Points

**Figure 4:**
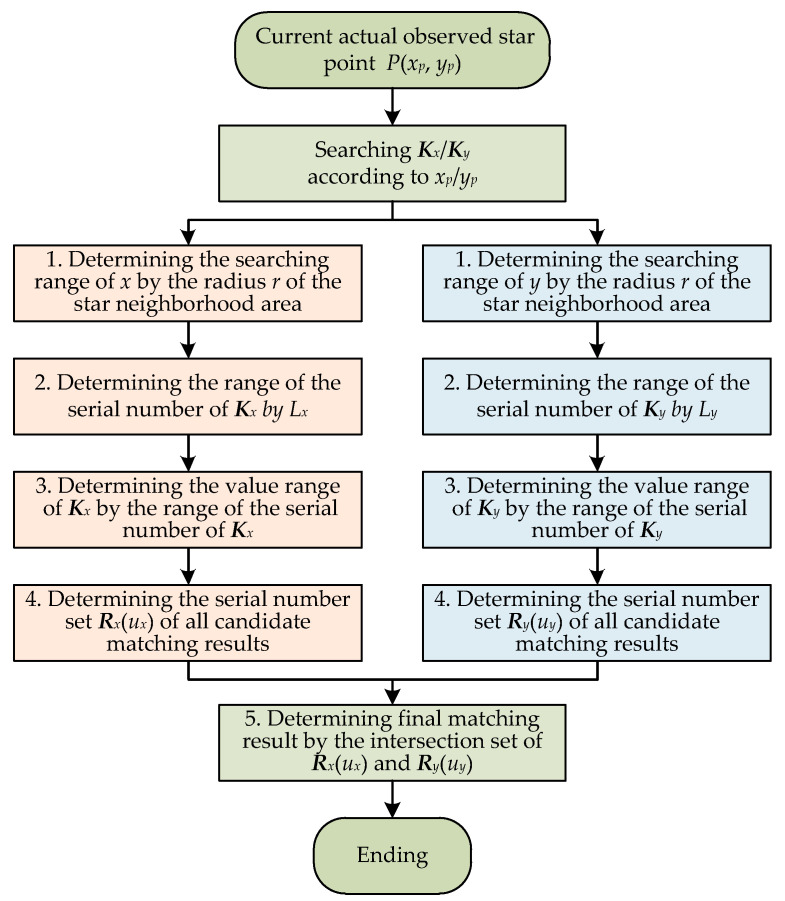
Flow chart of the matching of the observed star points.

Supposing that star point *P* is the current actual observed star point and its centroid information is (*x_p_*, *y_p_*), the fast matching process of star point *P* based on the DKVLUT is shown in Figure 4. Both the *x*-coordinate and the *y*-coordinate, i.e., *x_p_* and *y_p_*, are searched in ***K****_x_* and ***K****_y_*, respectively, and thus, the intersection of the above two search results is the final matching result of star point *P*. Since the searching processes of *x_p_* and *y_p_* are exactly the same, the following deduction will only take the searching process of *x_p_* as an example. 

Let star point *P*’ represent the final matching result of star point *P*; the *x*-coordinate range of *P*’ can be determined as [*x_a_*, *x_b_*] = [*x_p_* − *r*, *x_p_* + *r*], where *r* is the radius of the star neighborhood area. By utilizing the fitting line, *L_x_*, the range of the serial number of ***K****_x_*, [*i_a_*, *i_b_*], which is corresponding to the above *x*-coordinate range, [*x_a_*, *x_b_*], can be calculated as follows:

(4) $$i_{a}=\left\lfloor \frac{x_{a}-b_{1}}{a_{1}}\right\rfloor ,i_{b}=\left\lceil \frac{x_{b}-b_{1}}{a_{1}}\right\rceil ,$$

where⎿*x*⏌represents the largest integer less than or equal to *x*, and⎾*x*⏋represents the smallest integer greater than or equal to *x*. Subsequently, according to [*i_a_*, *i_b_*], the value range of ***K****_x_*, [*k_x_start_*, *k_x_end_*], can be determined as follows:

(5) $$k_{x\_start}=K_{x}(i_{a}),k_{x\_end}=K_{x}(i_{b}).$$

It should be noticed that the value range of ***K****_x_*, [*k_x_start_*, *k_x_end_*], is also the value range of ***U****_x_* in Table 1. That is to say, the value range of ***U****_x_*, [*u_xa_*, *u_xb_*], is equal to [*k_x_start_*, *k_x_end_*]. At this time, all candidate serial numbers of star point *P*’, which are only in terms of *x_p_*, can be quickly searched and ultimately determined as ***R****_x_*(*u_x_*), and all candidate *x*-coordinates corresponding to ***R****_x_*(*u_x_*) are ***S****_x_*(*u_x_*), where *u_x_* ∈ [*u_xa_*, *u_xb_*].

Meanwhile, for the *y*-coordinate of star point *P*, *y_p_*, the same searching process as that of the *x*-coordinate mentioned above can be adopted. It can also be deduced that the value range of ***K****_y_*, [*k_y_start_*, *k_y_end_*], is equal to the value range of ***U****_y_*, [*u_ya_*, *u_yb_*]. Similarly, all candidate serial numbers of star point *P*’, which are only in terms of *y_p_*, can also be quickly searched and ultimately determined as ***R****_y_*(*u_y_*), and all candidate *y*-coordinates corresponding to ***R****_y_*(*u_y_*) are ***S****_y_*(*u_y_*), where *u_y_* ∈ [*u_ya_*, *u_yb_*]. Obviously, the final matching serial number of star point *P*’, *r_p_*, should satisfy both ***R****_x_*(*u_x_*) and ***R****_y_*(*u_y_*), which can be described as follows:

(6) $$\begin{array}{l} r_{p}\in R_{P}=[R_{x}(u_{x})\cap R_{y}(u_{y})],\\ (u_{x}\in [u_{xa},u_{xb}],\;u_{y}\in [u_{ya},u_{yb}]), \end{array}$$

where ***R****_P_* is the intersection set of ***R****_x_*(*u_x_*) and ***R****_y_*(*u_y_*). Based on Equation (6), the following can be concluded:

(a) If ***R****_P_* is an empty set, no star point *P*’ can be found as the final matching result of the current observed star point *P*.
(b) If ***R****_P_* is a one-element set, the unique star point *P*’ can be found as the final matching result of the current observed star point *P*.
(c) If ***R****_P_* is a multi-element set, more than one matching result can be found, and the nearest one among them is chosen as star point *P*’—the final matching result of the current observed star point *P*.

To sum up, after the successful establishment of the DKVLUT, fast matching between all observed star points with disordered distribution and all predicted star points with sequential distribution can be realized by repeating the above matching process. In order to make readers understand clearly, examples on the establishment of the DKVLUT and the matching of the observed star points are detailed in Appendix A. 

## 4. Simulation and Analysis

In order to quantitatively evaluate the performance of the proposed method, both the proposed method in Section 3 and the existing method in Section 2 were applied as the star matching process of a multi-exposure star image, and the simulation results were compared and analyzed. The computer environment used in the simulation experiment was as follows: Win7 system, 2.6 GHz main-frequency CPU, four-core processor and MATLAB R2014a simulation platform. Based on the star catalog of the Smithsonian Astrophysical Observatory (SAO), navigation stars whose magnitude is less than or equal to 6.0 M were selected for the simulation experiment. Other simulation parameters of the star tracker are shown in Table 2. 

**Figure 5:**
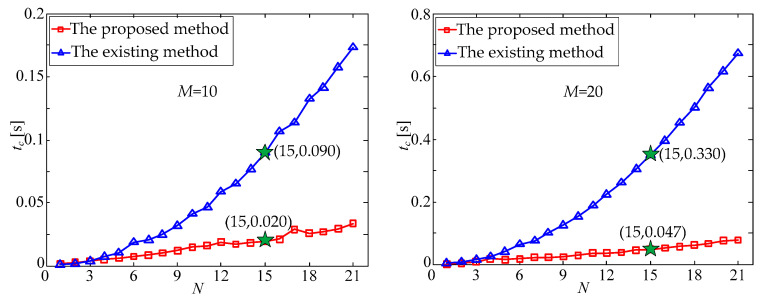

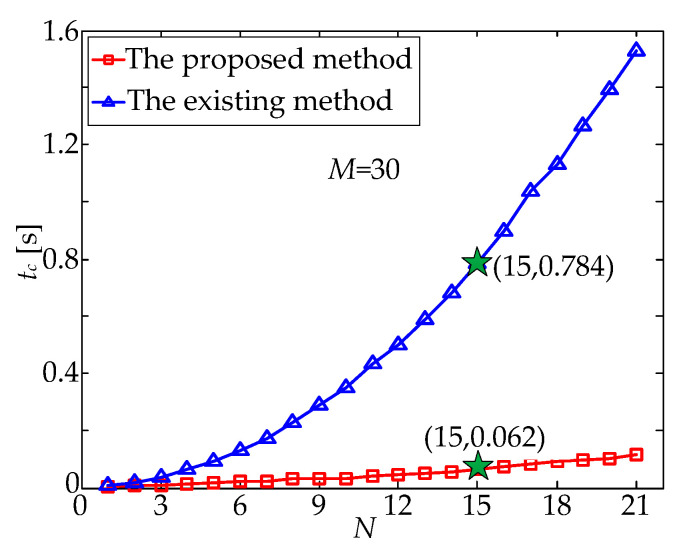
Processing time, *t_c_*, versus multi-exposure times, *N*.

In the experiment, the random number method was firstly used to generate the attitude matrix of the star tracker. Subsequently, the MEIA was applied to generate the simulated multi-exposure star image. The multi-exposure star points were directly set as predicted star points; by contrast, the multi-exposure star points with random noise (mean value *μ* = 0 and standard deviation *σ* = 1.0 pixel) were set as the observed star points so as to simulate the actual situation. Finally, under the above experimental conditions, both the proposed method and the existing method were applied as the star matching process between observed and predicted star points. With the increase in multi-exposure times, *N*, the processing time, *t_c_*, of the two matching methods changed, as shown in Figure 5. It should be noted that the processing time of the proposed method in the simulation included both the establishing time of the DKVLUT and the matching time of the observed star points.

It can be seen from Figure 5 that with the increase in *N*, the processing time, *t_c_*^,^, of the existing matching method increased significantly; in contrast, the processing time, *t_c_*, of the proposed method increased with a relatively slow speed. Taking *N* = 15 and *M* = 30 as an example, the processing time, *t_c_*, of the existing method was 0.784 s, while that of the proposed method was only 0.062 s. 

**Figure 6:**
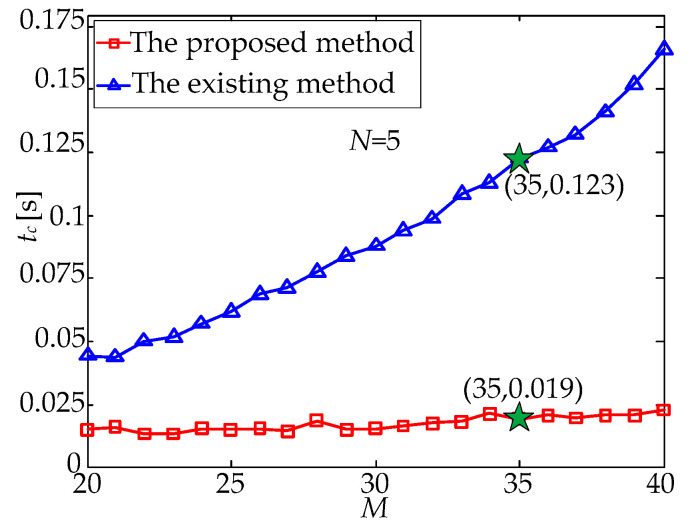

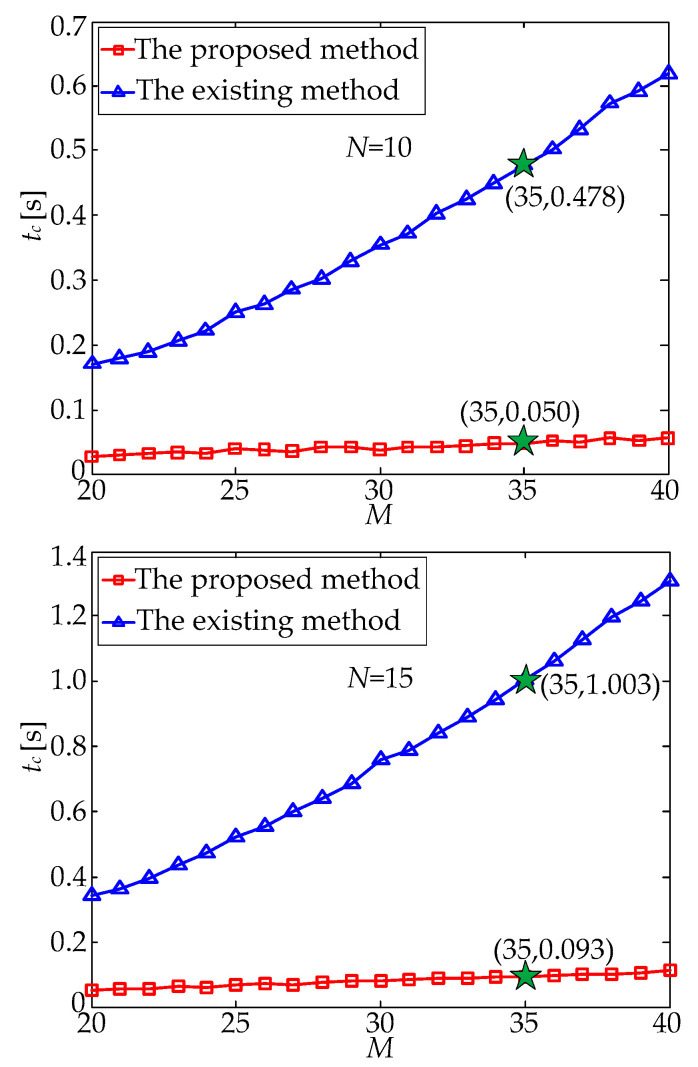
Processing time, *t_c_*, versus the number, *M*, of navigation stars.

To further verify the performance of the proposed method, the multi-exposure time was fixed, and the random noise of the centroid information of the observed star points was still set at a mean value *μ* = 0 and standard deviation *σ* = 1.0 pixel. Then, the random number method was used to generate different attitude matrices, and the number of navigation stars in the field of view (FOV) was usually different at different attitude matrices. At this time, a dataset with 20–40 navigation stars in the FOV was selected to repeat the above experimental process; that is, the star matching process was performed by using the existing method and the proposed method. As the number of navigation stars, *M*, increased gradually, the processing time, *t_c_*, of the two matching methods changed as shown in Figure 6. It can be seen from Figure 6 that with the increase in *M*, the processing time, *t_c_*, of the existing method increased significantly; in contrast, the processing time of the proposed method increased with a relatively slow speed. Taking *M* = 35 and *N* = 15 as an example, the processing time of the existing method was 1.003 s, while that of the proposed method was only 0.093 s.

With the gradual increase in *N* or *M*, the total number *W* = *MN* of star points in the multi-exposure star image increases accordingly, thus resulting in the increased complexity of the star matching process, and the processing time of the existing method increases sharply. In contrast, although the processing time of the proposed method is also increased, the increasing speed is relatively slow. To sum up, the processing time of the proposed method is approximately reduced by an order of magnitude compared with that of the existing method, which is shown in Figure 5 and Figure 6. 

**Figure 7:**
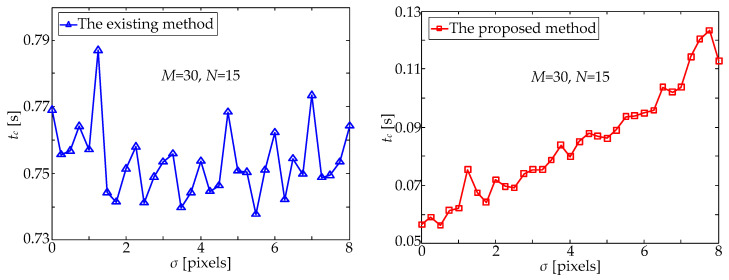
Processing time, *t_c_*, versus the standard deviation, σ.

In practice, due to star centroiding errors as well as star prediction errors, there is always a certain deviation between observed and predicted star points. Considering this, some random noises are added to simulate the actual situation, and the mean value *μ* and the standard deviation σ are two key parameters of the random noises. Nevertheless, it should be noticed that the position of the predicted star usually fluctuates around the actual star position. That is to say, the actual deviation usually obeys the distribution with a mean value of zero. Therefore, in order to quantitatively analyze the influence of the above deviation on the processing time of the two star matching methods, random noises with a mean value *μ* = 0 and different standard deviation values, *σ*, were added to the centroid information of the observed star points. With the increase in *σ*, the processing time of the two matching methods changed as shown in Figure 7. 

It can be seen from Figure 7 that the existing method is not sensitive to the position deviation between the observed and predicted star points. The existing method directly matches the distance between the observed and predicted star points and, thus, selects the nearest predicted star point as the matching result of the observed one, which has little relationship with that position deviation. In contrast, the proposed method is more sensitive to the above-mentioned position deviation, and its processing time, *t_c_*, gradually increases with the increase in standard deviation, *σ*, which is shown in Figure 7. The increase in position deviation directly leads to the increase in the radius, *r*, of the star neighborhood area, thus expanding the range of candidate serial numbers, [*i_a_*, *i_b_*], determined by Equation (4) and, finally, increasing the number of candidate star points determined by Equation (6). At this time, the existing matching method must be used to select the matching result among all candidate star points, which inevitably results in the increase in the processing time. Therefore, when applying the proposed method to star matching, it is quite necessary to improve the accuracy of star prediction and reduce the radius, *r*, of the star neighborhood area. Fortunately, when the star position is predicted by using the attitude information of the star tracker combined with the complete motion parameters (i.e., the angular velocity and the angular acceleration), the accuracy of star prediction can be significantly improved, and then, the radius, *r*, of the star neighborhood area can be effectively reduced, which is very beneficial to the application of the proposed fast star matching method in this paper. The above prediction method has been described in detail in a previous published paper [26] and, thus, will not be repeated here. 

**Figure 8:**
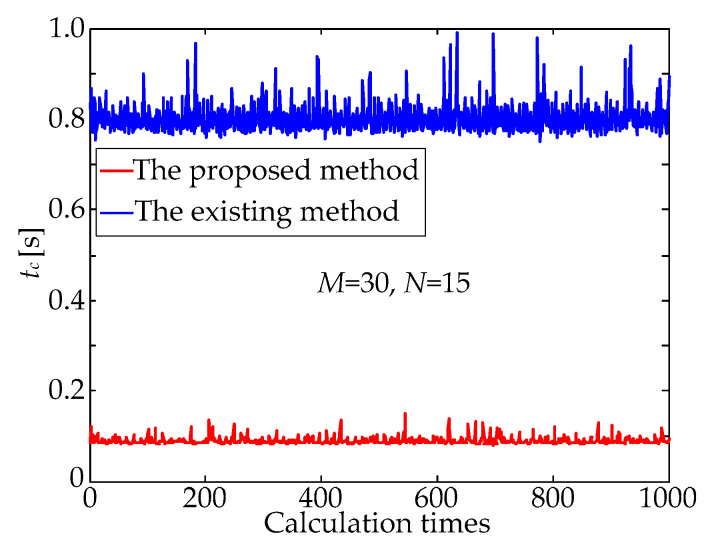
Processing time, *t_c_*, versus the calculation times.

Lastly, a large number of repeated simulation experiments were carried out to verify the calculation stability of the proposed method. The time of the experiments was set as 1000, and the random noise of the centroid information of the observed star points was still set as mean value *μ* = 0 and standard deviation *σ* = 1.0 pixel. The processing time, *t_c_*, of the two matching methods is described in Figure 8, and the statistical characteristics of the processing time, *t_c_*, can be seen in Table 3. As shown in Figure 8 and Table 3, the standard deviation of the proposed method is about a quarter of that of the existing method, which means the calculation stability of the former is better than that of the latter. Meanwhile, the mean value of the proposed method is approximately reduced by an order of magnitude compared with that of the existing method, which means that the former is remarkably faster than the latter. 

## 5. Night Sky Experiment

**Figure 9:**
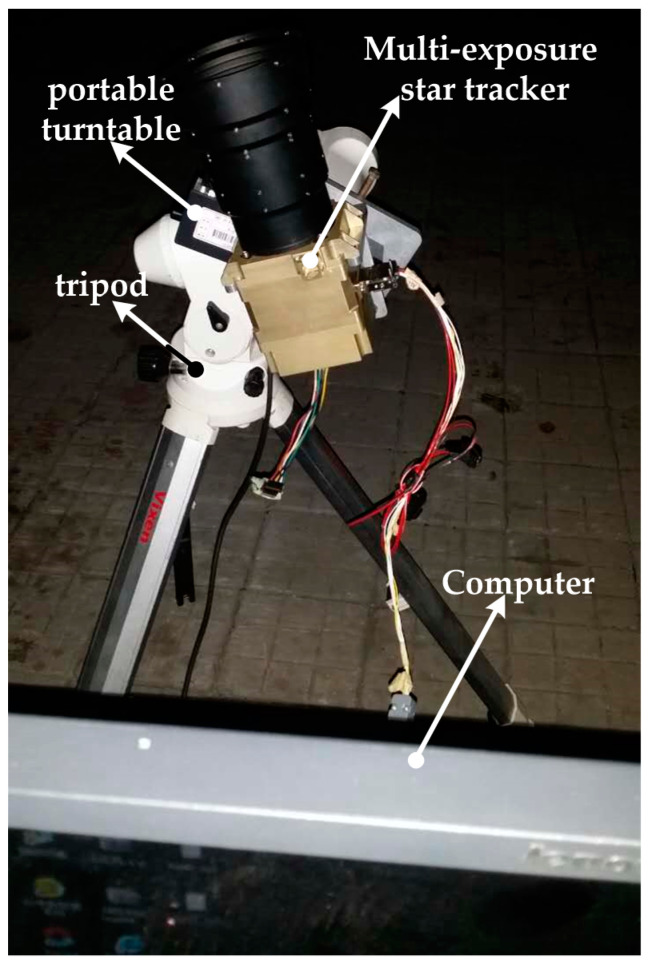
Experimental equipment.

A real night sky experiment was conducted to further validate the feasibility and effectiveness of the proposed method. Figure 9 shows all of the experimental equipment, which mainly included the multi-exposure star tracker, a portable high-precision turntable, a tripod, a computer, etc. The multi-exposure star tracker was solidly mounted on the portable high-precision turntable and, thus, could simultaneously rotate with the latter. 

During the experiment, the angular velocity of the turntable gradually increased from 0 to 20 °/s, while the multi-exposure times, *N*, changed accordingly from 1 to 21. Meanwhile, the number of navigation stars, *M*, changed with the variation of the FOV of the star tracker. When choosing two groups of parameters within the experiment, the two correlated star images were uploaded to the computer, which was the same computer used in Section 4 by the star tracker. Under the same computing platform and the same star image, both the existing method and the proposed method were adopted to fulfill the matching process, and their processing times are listed in Table 4. It should be noted that the processing time of the proposed method in Table 4 still includes both the establishing time of the DKVLUT and the matching time of the observed star points, which is the same as the simulation part. 

The experimental results indicate that the processing time of the existing method is about 5~8 times as long as that of the proposed method, which is basically consistent with the simulation results mentioned in Section 4 and, thus, further demonstrates the feasibility and effectiveness of the proposed method.

## 6. Discussion

As described above, both simulation and night sky experiments were conducted, and the respective results are provided. In the simulation, the processing time of the two matching methods changes with the increase in multi-exposure times, *N*, or the number of navigation stars, *M*. The processing time, *t_c_*, of the proposed method is approximately reduced by an order of magnitude compared with that of the existing method, which indicates that the proposed method is notably faster than the existing one. Besides, when the random noises increased gradually, the simulation results show that the proposed method is more sensitive than the existing one is, which indicates that random noises should be reduced before the proposed method is adopted. Lastly, the results of repeated simulation experiments also show that the proposed method has a much smaller mean value and standard deviation than the existing one, which indicates that the calculation stability of the former is better than that of the latter. In the night sky experiment, the processing time of the proposed method was about 5~8 times shorter than that of the existing method, which further demonstrates the feasibility and effectiveness of the proposed method.

In some situations, it seems that the single *K*-vector (e.g., *x*-axis) is sufficient to match the predicted and observed stars properly, because the distance calculation seems to be executed only for a few stars. Unfortunately, in other situations, the distance calculation would increase to a huge amount of computation if the angle between the motion direction of stars and the *y*-axis is relatively small (in the extreme state, the above angle can be zero). In this case, many distance calculations would be needed if the single *K*-vector-based method is adopted, and that is why the DKVLUT-based method is proposed in this paper. 

As for the benefits of the DKVLUT-based method, they can be divided into two advantages. Firstly, the proposed method itself remarkably speeds up the matching process of two association datasets. Other similar applications can also adopt the proposed method. Secondly, the proposed method successfully overcomes the new bottleneck, the star matching step, which is caused by MEIA. That is to say, after adopting the proposed method, the star matching step is not the bottleneck any more, and thus, the MEIA can be used to improve the update rate, which allows crucial progress for the development of star tracker technology.

Furthermore, it should be noted that stray light (e.g., sunlight) may also have some influence on the proposed method. When the star tracker is working under highly dynamic conditions, its FOV will quickly sweep through a large number of sky areas, thus inevitably resulting in more stray light entering the imaging field of the star tracker. In this case, the number of false star targets caused by the stray light will increase heavily, which will adversely affect the performance of the proposed method. Therefore, improvements in the robustness against stray light still needs to be made in future research.

## 7. Conclusions 

The MEIA proposed in the earlier study can be used to improve the attitude update rate by *N* times and, thus, is quite effective for the development of high-update-rate star trackers. Unfortunately, when the existing star matching method is adopted to match the observed and predicted stars in the MEIA, the matching time significantly increases with the increase in multi-exposure times, *N*, or the number of navigation stars, *M*, in the FOV, which severely affects the MEIA’s performance. Therefore, a fast star matching method based on a DKVLUT for multi-exposure star trackers was proposed in this paper. The principle of the proposed method has been described in detail, including the establishment of the DKVLUT as well as the matching process of the observed and predicted star points. Subsequently, both the proposed method and the existing method were simulated, and the simulation results were compared and analyzed. Taking *N* = 15 and *M* = 30 as an example, the processing time, *t_c_*, of the existing method was 0.784 s, while that of the proposed method was only 0.062 s. The simulation results show that under the same experimental conditions, the processing time of the proposed method is reduced by approximately an order of magnitude compared with that of the existing method. Finally, real night sky experiments were conducted to further evaluate the proposed method as well as the existing method, and the results also demonstrate the feasibility and effectiveness of the proposed method. 

## Figures and Tables

**Table 1:** Information of all three vectors.

*U_x_*	*K_x_*	*S_x_*	*R_x_*
1	0	***S****_x_*(1)	***R****_x_*(1)
2	***K****_x_*(2)	***S****_x_*(2)	***R****_x_*(2)
3	***K****_x_*(3)	***S****_x_*(3)	***R****_x_*(3)
…	…	…	…
*l_s_* − 1	***K****_x_*(*l_s_* − 1)	***S****_x_*(*l_s_* − 1)	***R****_x_*(*l_s_* − 1)
*l_s_*	*l_s_*	***S****_x_*(*l_s_*)	***R****_x_*(*l_s_*)

**Table 2:** Simulation parameters of the star tracker.

Parameter	Value
Field of View (FOV) (°)	20 × 20
Focal Length *f* (mm)	31.94
Resolution *A* × *A* (pixels)	2048 × 2048
Pixel Size *a* × *a* (μm)	5.5 × 5.5

**Table 3:** Statistical characteristics of the processing time, *t_c_*.

Method	Mean Value of *t_c_*	Standard Deviation of *t_c_*
The existing method	0.090 s	0.008 s
The proposed method	0.799 s	0.030 s

**Table 4:** Processing time *t_c_* of two matching method.

Method	*N* = 12, *M* = 24	*N* = 16, *M* = 28
The existing method	*t_c_* = 0.245 s	*t_c_* = 0.588 s
The proposed method	*t_c_* = 0.046 s	*t_c_* = 0.072 s

## Data Availability

Not applicable.

## Appendix A

In order to clearly clarify the establishment process of the DKVLUT, a simple example of six total predicted stars is expressed as follows. The coordinates of six predicted stars are supposed as (506.4, 45.6), (23.2, 347.9), (105.8, 78.5), (1023.2, 732.6), (243.1, 381.5) and (132.7, 176.3). According to Figure 3, the above coordinates should be divided into two parts, i.e., *x*-coordinate and *y*-coordinate vectors. When taking the *x*-coordinate vector as an example, the establishing process of ***K****_x_* includes four steps in total.

Firstly, according to the *x*-coordinate vector of [506.4, 23.2, 105.8, 1023.2, 243.1, 132.7]^T^, ***S****_x_* can subsequently be rearranged as a vector of [23.2, 105.8, 132.7, 243.1, 506.4, 1023.2]^T^, and ***R****_x_* can, thus, be a vector of [2, 3, 6, 5, 1, 4]^T^ corresponding to ***S****_x_*.

Secondly, according to Equation (1), *D_x_* can be calculated as follows:

(A1) $$D_{x}=\frac{S_{x}(l_{s})-S_{x}(1)}{l_{s}-1}=\frac{1023.2-23.2}{6-1}=200.$$

Therefore, the fitting line *L_x_* can be determined by Equations (2) and (3) as follows:

(A2) $$h_{x}=240m_{x}-316.8,$$

Subsequently, according to the above calculation rules, ***K****_x_* is calculated as a vector of [0, 3, 4, 5, 5, 6]^T^.

Lastly, the lookup table of ***K****_x_* is described in Table A1. Similarly, the lookup table of ***K****_y_* is also listed in Table A1.

**Table A1:** Example of a DKVLUT.

*U_x_*	*K_x_*	*S_x_*	*R_x_*	*U_y_*	*K_y_*	*S_y_*	*R_y_*
1	0	23.2	2	1	0	45.6	1
2	3	105.8	3	2	2	78.5	3
3	4	132.7	6	3	3	176.3	6
4	5	243.1	5	4	5	347.9	2
5	5	506.4	1	5	5	381.5	5
6	6	1023.2	4	6	6	732.6	4

In order to intuitively understand the matching process of the observed star points, an example of an observed star point, whose coordinates are supposed as (246.7, 378.5), is conducted based on the DKVLUT in Table A1.

According to Figure 4, the matching process includes five steps:

(1) By setting *r* = 5 to ensure that the star neighborhood area is big enough to contain the target matching result, the *x*-coordinate and *y*-coordinate ranges can be determined as [*x_p_* − *r*, *x_p_* + *r*] = [ 241.7, 251.7] and [*y_p_* − *r*, *y_p_* + *r*] = [ 373.5, 383.5], respectively.
(2) According to Equation (4), [*i_xa_*, *i_xb_*] can be determined as follows:

(A3) $$i_{xa}=\left\lfloor \frac{x_{a}-b_{1}}{a_{1}}\right\rfloor =2,i_{xb}=\left\lceil \frac{x_{b}-b_{1}}{a_{1}}\right\rceil =3.$$

Similarly, [*i_ya_*, *i_yb_*] can be determined as [3,4].

(3) According to Equation (5) and Table A1, [*k_x_start_*, *k_x_end_*] can be determined as follows:

(A4) $$k_{x\_start}=K_{x}(i_{a})=3,k_{x\_end}=K_{x}(i_{b})=4.$$

Similarly, [*k_y_start_*, *k_y_end_*] can be determined as [3, 5].

(4) [*u_xa_*, *u_xb_*] is equal to [*k_x_start_*, *k_x_end_*] = [3, 4], and thus, the serial number set of all candidate matching results, ***R****_x_*(*u_x_*), can be determined as {6, 5}.

Similarly, [*u_ya_*, *u_yb_*] is equal to [*k_y_start_*, *k_y_end_*]= [3, 5], and thus, the serial number set of all candidate matching results, ***R****_y_*(*u_y_*), can be determined as {6, 2, 5}.

(5) According to Equation (6), the intersection set ***R****_P_* can be determined as {6, 5}. At this time, both star 6 and star 5 are selected to calculate the distance, and the nearer star 5, (243.1, 381.5), should be the final matching result.
